# Serial detection of circulating tumour cells by reverse transcriptase-polymerase chain reaction assays is a marker for poor outcome in patients with malignant melanoma

**DOI:** 10.1186/1471-2407-6-266

**Published:** 2006-11-15

**Authors:** Giuseppe Palmieri, Sabrina MR Satriano, Mario Budroni, Antonio Cossu, Francesco Tanda, Sergio Canzanella, Corrado Caracò, Ester Simeone, Antonio Daponte, Nicola Mozzillo, Giuseppe Comella, Giuseppe Castello, Paolo A Ascierto

**Affiliations:** 1 Istituto di Chimica Biomolecolare-Consiglio Nazionale Ricerche, Li Punti-Sassari, Italy; 2 Istituto Nazionale Tumori "Fondazione Pascale", Napoli, Italy; 3 Centro Multizonale di Osservazione Epidemiologica, Azienda U.S.L. 1, Sassari, Italy; 4 Servizio di Anatomia Patologica, Azienda U.S.L. 1, Sassari, Italy; 5 Associazione House Hospital Onlus, Napoli, Italy

## Abstract

**Background:**

Detection of circulating malignant cells (CMCs) through a reverse transcriptase-polymerase chain reaction (RT-PCR) assay seems to be a demonstration of systemic disease. We here evaluated the prognostic role of RT-PCR assays in serially-taken peripheral blood samples from patients with malignant melanoma (MM).

**Methods:**

One hundred forty-nine melanoma patients with disease stage ranging from I to III were consecutively collected in 1997. A multi-marker RT-PCR assay was used on peripheral blood samples obtained at time of diagnosis and every 6 months during the first two years of follow-up (total: 5 samples). Univariate and multivariate analyses were performed after 83 months of median follow-up.

**Results:**

Detection of at least one circulating mRNA marker was considered a signal of the presence of CMC (referred to as PCR-positive assay). A significant correlation was found between the rate of recurrences and the increasing number of PCR-positive assays (P = 0.007). Presence of CMC in a high number (≥2) of analysed blood samples was significantly correlated with a poor clinical outcome (disease-free survival: P = 0.019; overall survival: P = 0.034). Multivariate analysis revealed that presence of a PCR-positive status does play a role as independent prognostic factors for overall survival in melanoma patients, adding precision to the predictive power of the disease stage.

**Conclusion:**

Our findings indicated that serial RT-PCR assay may identify a high risk subset of melanoma patients with occult cancer cells constantly detected in blood circulation. Prolonged presence of CMCs seems to act as a surrogate marker of disease progression or a sign of more aggressive disease.

## Background

The poor prognosis of patients with malignant melanoma (MM) is mostly due to the high frequency of distant dissemination of the disease. Considering the small size of most primary melanomas, the metastatic potential of MM is considerably greater than that of most other solid tumours [1].

The relationship between presence of circulating cancer cells and development of secondary disease is not fully understood. Detection of circulating malignant cells (CMCs) is actually aimed to the identification of either an earlier marker of tumour progression or a phenotypic feature of more aggressive disease that might change treatment options [2]. Detection of CMCs in peripheral blood of MM patients at early-stage disease could indicate a systemic dissemination of the tumour cells and, thus, a higher risk of developing metastasis [3].

Reverse transcriptase-polymerase chain reaction (RT-PCR) can detect a single specific messenger RNA (mRNA) in a mixed cell population; thus, it can be a sensitive method for identification of circulating tumour cells [4-7]. Tyrosinase (TYR), an enzyme that is involved in the melanin biosynthesis pathway [8], is the marker most frequently used to detect the presence of CMC in melanoma patients; however, its clinical usefulness is highly debated [9-13]. To improve sensitivity and specificity of the procedure, we and others have previously proposed a multi-marker RT-PCR assay, including melanA/MART1 in addition to tyrosinase (the use of TYR mRNA as unique marker could be of limited value in the management of MM patients, due to the heterogeneity of TYR expression in melanomas) [14-18]. Such a multi-marker RT-PCR assay has been demonstrated to really improve the detection of melanoma-associated transcripts in peripheral blood of patients who have undergone radical surgery [7,14,15]. Detection of minimal residual disease at the time of diagnosis by RT-PCR seems to be correlated with the initial clinical stage; however, our group has demonstrated that it does not add any power to the predictive value of the disease stage [19].

We here investigated whether serial detection of circulating melanoma-associated markers by performing consecutive multi-marker RT-PCR assays on peripheral blood samples obtained during patients' follow-up may improve prognostic prediction, commonly based on pathological and clinical factors. Moreover, the current study was designed to examine the impact of longer duration of follow-up on the prognostic relevance of such a molecular assay in the assessment of clinical outcome among melanoma patients.

## Methods

### Sample collection

The study was conducted with patients referring to the National Cancer Institute of Naples between January 9, 1997 and December 16, 1997. Patients were consecutively collected and considered eligible if they had a histological diagnosis of cutaneous malignant melanoma (performed either inside or outside the Institute; in the latter case, slides were reviewed by internal pathologists). Eight out of 157 enrolled patients were excluded from the series since they were early lost in follow-up (in a period ranging from 9 to 14 months).

For the 149 patients included into the study, clinical stage was documented at the time of enrollment and prospectively followed-up. Patients with no clinical evidence of regional or metastatic disease underwent the sentinel lymph node (SLN) dissection. Histopathologic examination was performed on each formalin-fixed paraffin-embedded SLN using hematoxylin/eosin staining and immunohistochemistry (IHC) of adjacent sections. For IHC, 4-μm-thick sections were evaluated by using antibodies against HMB-45 and S-100 proteins. All stage III patients received the same adjuvant therapy with low dose interferon alpha (as we previously described [20]). Disease stage has been codified by clinicians in charge of the patients, according to the recent American Joint Committee on Cancer (AJCC) guidelines [21].

For collection of baseline peripheral blood samples (time 0; sample 1), patients (stage I to III) were eligible if no more than two weeks had elapsed from the surgical treatment. All patients were visited every six months after the diagnosis. During the first two years of follow-up, peripheral blood samples were taken at 6, 12, 18, and 24 months after diagnosis (times 1, 2, 3, and 4; samples 2, 3, 4, and 5; respectively) and addressed to the RT-PCR screening. Death before the third blood sample (within 12 months after diagnosis) was an exclusion criterion; no patients was excluded for such a criterion. No clinical decision was made based on the result of the RT-PCR assay.

At each follow-up time, a clinical history, physical examination, cell blood count, and blood biochemistry were performed. Instrumental assessments were performed if clinically indicated. Patients were informed about aims and limits of the study; peripheral blood samples for RT-PCR analysis were obtained with their written consent. The study was reviewed and approved by the ethical review boards of both the National Tumor Institute of Naples and the University of Sassari.

### Sample Preparation and RT-PCR assay

Nucleated cells from peripheral blood were processed in order to isolate total RNA, using standard procedures.

In our previous report, a multiple-marker RT-PCR assay (including tyrosinase, p97, and MelanA/MART1 markers) was tested for its sensitivity and specificity in detecting circulating melanoma-associated mRNAs [15]. Analysis of total cellular RNA obtained from peripheral blood of 235 patients with either localized or metastatic MM as well as of 41 negative controls (20 healthy subjects and 21 patients with different types of cancer) demonstrated that maximal values of sensitivity and specificity were reached by tyrosinase and MelanA/MART1 [15]. In the present study, we therefore decided to use such two markers (primer sequences and protocols for RT-PCR assay were as previously described [15]).

Integrity of RNA was determined by performing parallel RT-PCR reaction using primers specific for the housekeeping gene GAPDH [15]. Samples that failed to amplify products for GAPDH RNA were considered non-informative and discarded. In each RT-PCR assay, final products were separated by electrophoresis on 2% agarose gel and analyzed by direct visualization after ethidium bromide staining. Specificity of the RT-PCR products was assessed by Southern blot analysis, as previously described [15]. Samples were considered positive when a PCR product was detected by either direct visualization or blot analysis.

### Statistical Analysis

To assess whether the detection of CMC in peripheral blood samples serially taken from MM patients could have prognostic value, the following criteria were used. PCR-based amplification of at least one mRNA marker (tyrosinase, MelanA/MART1, or both) in a peripheral blood specimen was considered a signal of the presence of CMC (referred to as PCR-positive assay). Presence of CMC was used as a binary (no/yes) explanatory variable.

Univariate associations between PCR-based markers and clinico-pathological variables [AJCC stage (I to III), Breslow thickness of primary tumour [22], Clark level of invasion (I to V) [23], sex, age at diagnosis] were investigated by chi-square test. The Cox regression model was performed using the raw mortality and the tumour specific mortality as a censoring. The time of overall survival was expressed in months. In all statistical analyses, P < 0.05 was considered significant. All analyses were performed with the statistical package SPSS/7.5 per Windows.

## Results

A consecutive cohort of 149 patients with histologically-proven diagnosis of MM from January 9, 1997 to December 16, 1997 was studied. Patients were prevalently females, with a median age of 48 years (range 16–85). According to the new AJCC stage classification, almost the half of the patients had stage I disease (72; 48%), and one-third had stage II (49; 33%). Overall, patients without lymph node metastases (stages I to II) accounted for 81% of the series (Table 1).

Detection of circulating mRNA markers (tyrosinase, MelanA/MART1, or both) in a blood sample was considered a signal of the presence of CMC (referred to as PCR-positive assay). During the first two years after diagnosis, peripheral blood samples from MM patients undergoing follow-up visits every six month (times 6, 12, 18, and 24 months) were screened for CMC by RT-PCR assays. In Table 2, total number of PCR-positive assays (ranging from 0, in patients with all five blood samples negative for mRNA markers, to 5, in patients with all five blood samples positive for mRNA markers) was compared to the disease stage. No statistical association between positive RT-PCR assays and the stage of disease was observed.

As of December 2005, 49 patients (33%) have suffered progression (site of recurrence: 27 regional lymph nodes, 6 distant lymph nodes, 7 skin (*in transit *metastases), 6 visceral sites, 3 brain); 28 patients (19%) have died of melanoma. For the entire series of 149 patients included into the study, median follow-up was 83 months (range, 24–93). Four patients recurred after 20 months; blood drawing at month 24 (sample 5) was anyhow performed and such patients were not excluded from the study. No patient in our series died within 24 months from diagnosis.

At univariate analysis the presence of PCR-positive assays had a significant predictive value. As shown in Table 3, we compared the total number of PCR-positive assays (regardless the timing of the blood sampling) with either the rate of disease recurrences or the median disease-free survival (DFS) and the median overall survival (OS). A significant correlation was found between the rate of recurrences and the increasing number of PCR-positive assays [11 (17%) relapses in 64 cases with 0–1 PCR-positive assay vs. 38/85 (45%) cases with ≥2 PCR-positive assays (P = 0.007)] (Table 3). While no significant difference in DFS and OS was observed for MM patients with ≤2 positive RT-PCR assay (median DFS: 81 months; median OS: 83 months), presence of CMC in ≥3 peripheral blood samples was significantly correlated with a poor clinical outcome [median DFS: 73 months (P = 0.019); median OS: 76 months (P = 0.034)] (Table 3).

Considering the timing of PCR results, a correlation between the rate of recurrences and the variation of the CMC status during follow-up was inferred. In Table 4, patients were classified according to the RT-PCR results obtained at and after baseline blood drawing. A quite low relapse rate was observed in patients with all five RT-PCR assays negative as well as in those who went from positive in initial assay to negative in remaining assays (altogether, 4/33; 12%) (Table 4). On the other hand, frequency of relapses were much higher in patients with positive RT-PCR assays during follow-up, regardless the baseline status (altogether, 45/116; 39%) (Table 4).

No statistical correlation between the RT-PCR status and Breslow thickness, Clark level of invasion, primary site, sex, or age at diagnosis was observed.

The Cox regression multivariate analysis was performed on the totality of patients in order to estimate the role of different disease parameters as well as of the RT-PCR status on clinical outcome in terms of overall survival. In addition to the expected variables represented by the primary tumor extension (as inferred by the Breslow thickness) (Table 5) or the more reliable and comprehensive stage of disease (Table 6), the presence of at least two PCR-positive assays remained a statistically independent prognostic factor for overall survival in patients with malignant melanoma (Tables 5 and 6).

## Discussions and Conclusion

In this paper, we showed that presence of circulating malignant cells (CMCs), assessed through a PCR-based detection of multiple mRNAs corresponding to melanoma-associated molecular markers (tyrosinase and MelanA/MART1), in a high fraction of peripheral blood samples serially obtained during follow-up visits, does play a role as independent predictive factor for clinical outcome.

Taking into consideration both the raw mortality and the tumour specific mortality as a censoring, survival data analysis allowed to assess that patients with presence of melanoma cells in at least two out of the five analyzed peripheral blood samples (referred to as PCR-positive assays), collected every six months during the first two years of follow-up, had a worse clinical outcome with a significantly shorter overall survival over a median follow-up of about seven years (83 months). Multivariate analysis revealed that such a high level (≥2) of PCR-positive assays does correlate with the overall survival in melanoma patients, thus adding precision to the predictive power of the stage of disease (see Tables 5 and 6).

Nevertheless, increasing number of PCR-positive assays in serially-taken peripheral blood samples was also found significantly associated with a higher rate of relapses (see Table 3). This seems to further suggest that such an approach could help in defining a high risk subset of melanoma patients.

A multiple-marker RT-PCR assay has been demonstrated to be more sensitive and specific than single-marker assay in detecting circulating melanoma metastases [7,14,15]. Previously, we observed that mRNA expression of tyrosinase and MelanA/MART1 was quite completely undetectable in peripheral blood of nonmelanoma controls [15], confirming that these two markers possess the highest specificity and reliability in detection of melanoma micro-metastases. In addition, existence of a statistically significant association between disease stage and presence of such tumour-associated mRNAs in peripheral blood as expression of CMCs has been widely demonstrated [14,15,19,24]. However, clinical significance and prognostic role of circulating mRNA markers is highly debated and not completely understood [2,11-19].

Mobilization of cancer cells from the site of the primary lesion and physical invasion of blood stream are among the earliest events of tumour progression, necessary, although not sufficient, to produce distant metastases [25]. Indeed, colonization of distant tissues and development of metastasis represent the result of a multi-step cascade of events occurring to cancer cells during tumour dissemination (i.e., viability in circulation, capability of exiting blood stream and starting tissue invasion, presence of adequate growth potential for metastasis formation) [26]. Detection of circulating melanoma cells could reasonably correspond to the identification of an early potential step of metastatic dissemination.

Majority of the published studies in which a multivariate analysis has been performed reported the strongest correlation of PCR-detected CMCs with the disease stage at the time of diagnosis, rather than as an independent prognostic factor [13,15,24,27-30]. Among them, studies with positive conclusions mostly reported very low rates of events for analysis (quite all investigations were focused on progression-free survival, which cannot completely substitute the value of overall survival analysis) [28-30]. Longer follow-up are thus required in order to observe a higher number of such events (with particular attention to the melanoma-related deaths for accurate calculation of the overall survival [31]), in order to infer a more appropriate evaluation of the clinical role of RT-PCR assays.

Considering such a correlation between presence of CMCs at the time of diagnosis and the initial stage of disease, one could speculate that detection of CMCs may represent a surrogate for clinical staging. However, our group has previously demonstrated that detection of melanoma-associated transcripts in peripheral blood of melanoma patients at the time of diagnosis by RT-PCR, when adjusted by disease stage, had no significant predictive value for clinical outcome [19]. Stage of disease remained the only prognostic factor known to correlate with time to recurrence and/or overall survival [19,21].

The results of the present study further suggest that metastatic cancer cells might be constantly present in blood circulation of a subset of melanoma patients, before the establishment of distant metastases. In this light, prolonged presence of melanoma cells in blood stream may indeed represent either a surrogate marker of disease progression or a sign of more aggressive disease, due to selection of viable cancer cells with better capacity to invade the target tissue and higher growth potential at the distant site (see Table 4). Supporting this hypothesis, another study defined the existence of changes in CMCs during interferon therapy among MM patients with more advanced disease (stages II-IV) [32]. Using multivariate analysis, these Authors demonstrated that patients who became CMC-negative during interferon therapy were significantly associated with better disease-free survival than those who remained or became positive during therapy [32]. In addition to the stage of disease as the most important prognostic factor, change in CMC status during a prolonged observation period may be indeed of prognostic value.

Dynamic studies, such as the present one, aimed to assess any modification of the circulating malignant cell status by RT-PCR assay throughout follow-up or treatment deserves further prospective investigations among patients with melanoma. A more extensive application of the real-time quantitative RT-PCR approach [33,34] may further improve the specificity of standard qualitative RT-PCR assays. This may help in establishing a detection threshold above which sensitivity for clinically significant micrometastases can be optimized and false-positives can be minimized.

Although more accurate approaches for detection and characterization of circulating melanoma are under evaluation, the results of our analysis strongly indicate that any future study evaluating molecular status will necessitate suitable long-term median follow-up (at least five years) to provide clinically relevant information and define outcome for patients with occult melanoma metastases. Prediction of metastatic potential remains one of the main goals to be pursued in order to better assess the risk subgroups of patients with malignant melanoma.

## Competing interests

The author(s) declare that they have no competing interests.

## Authors' contributions

GP participated in the design of the study, performed the molecular analyses, and drafted the manuscript. SMRS performed the molecular analyses. MB performed the statistical analysis. AC performed the data management. FT participated in the analysis and interpretation of data. SC participated in the patients' collection. CC participated in the patients' collection. ES participated in the patients' collection. AD participated in the patients' collection. NM participated in the design of the study. GCo participated in the design of the study. GCa participated in the design of the study. PAA conceived of the study, and participated in its coordination.

All authors read and approved the final manuscript.

## Pre-publication history

The pre-publication history for this paper can be accessed here:



## Figures and Tables

**Table 1:** Characteristics of patients

***Characteristics***	***Number of patients***	*%*
**Total analyzed**	**149**	
**Sex**		
Males	59	*40*
Females	90	*60*
**Median Age (years)**	**48**	
Range	16–85	
**AJCC stage**		
**IA**	**50**	*33*
**IB**	**22**	*15*
**IIA**	**36**	*24*
**IIB**	**12**	*8*
**IIC**	**1**	*1*
**III**	**28**	*19*
**Primary site**		
Head and Neck	**13**	*9*
Superior limbs	**23**	*15*
Inferior limbs	**47**	*32*
Trunk	**63**	*42*
Unknown	**3**	*2*

Staging was according to the recent American Joint Committee on Cancer (AJCC) guidelines. Data refer to the time of diagnosis.

**Table 2:** PCR-positive assays in serially-taken peripheral blood samples.

**AJCC STAGE **(patients)	***PCR-positive assays***						***No. of PDs *** *(%)*
	**0 **(%)	**1 **(%)	**2 **(%)	**3 **(%)	**4 **(%)	**5 **(%)	
**I **(72)	**6 **(8)	**30 **(42)	**22 **(31)	**9 **(12)	**4 **(6)	**1 **(1)	***12 *** *(17)*
**IA **(50)	**3 **(6)	**21 **(42)	**15 **(30)	**7 **(14)	**3 **(6)	**1 **(2)	***8 *** *(16)*
**IB **(22)	**3 **(14)	**9 **(41)	**7 **(32)	**2 **(9)	**1 **(4)	**0 **(0)	***4 *** *(18)*
**II **(49)	**4 **(8)	**10 **(20)	**19 **(39)	**12 **(25)	**2 **(4)	**2 **(4)	***17 *** *(35)*
**IIA **(36)	**2 **(5.5)	**8 **(22)	**14 **(39)	**9 **(25)	**2 **(5.5)	**1 **(3)	***11 *** *(31)*
**IIB-IIC **(13)	**2 **(15)	**2 **(15)	**5 **(39)	**3 **(23)	**0 **(0)	**1 **(8)	***6 *** *(46)*
**III **(28)	**4 **(14)	**6 **(21)	**8 **(29)	**6 **(21)	**3 **(11)	**1 **(4)	***20 *** *(71)*

**TOTAL **(149)	**14 **(9)	**46 **(31)	**49 **(33)	**27 **(18)	**9 **(6)	**4 **(3)	***49 *** *(33)*

PD, progression disease

**Table 3:** Correlation between total number of PCR-positive assays and rates of progression disease or median survivals

**No. of positive assays**	**No. of patients**	**No. of PDs **(%)	**median DFS **(months)	**median OS **(months)
**0**	**18**	**3 **(17)	**81**	**83**
**1**	**46**	**8 **(17)	**81**	**83**
**2**	**45**	**12 **(27)	**82**	**83**
**3**	**27**	**17 **(63)	**78**	**79**
**4–5**	**13**	**9 **(69)	**66**	**70**

PD, progression disease; DFS, disease free-survival; OS, overall survival

**Table 4:** Correlation between timing of PCR results and rates of progression disease

**PCR assay results**		**No. patients**	**No. of PDs **(%)
		
**Baseline**	**Follow-up**		
**negative**	**negative**	**18**	**3 **(17)
**negative**	**positive**	**56**	**19 **(34)
**positive**	**negative**	**15**	**1 **(7)
**positive**	**positive**	**60**	**26 **(43)

PD, progression disease

**Table 5:** Multivariate analysis for overall survival including primary tumour extension

Marker	**Hazard Ratio**	**95% CI**	P
Sex	1,185	0,49–2.83	0.702
Age	1.108	0.93–1.13	0.390
Breslow thickness	2.530	1.62–3.94	< 0.001
CMC status (0–1 vs ≥2 positive assays)	2.685	1.17–6.49	0.026

CI, confidence interval

**Table 6:** Multivariate analysis for overall survival including disease stage

Marker	**Hazard Ratio**	**95% CI**	P
Sex	1.013	0.46–2.19	0.973
Age	1.011	0.98–1.04	0.480
AJCC Stage (I-III)	2.907	1.24–6.81	0.014
CMC status (0–1 vs ≥2 positive assays)	2.468	1.12–6.23	0.037

CI, confidence interval
